# Lung ultrasound and the interstitial syndrome in tuberculosis endemic regions: illustrated insights from diverse clinical settings

**DOI:** 10.1016/j.jctube.2026.100637

**Published:** 2026-07-20

**Authors:** Ablo Prudence Wachinou, Matthew Fentress, Jacques Du Toit, Aboudou Hada, Gildas Agodokpessi, Seydina Alioune Beye, Yacouba Cissoko, Khadidia Ouattara, Shabani Majaliwa, Thomas Brahier, Noémie Boillat-Blanco, Véronique Suttels

**Affiliations:** a National Taching Hospital for Tuberculosis and Pulmonary Diseases (CNHU-PPC), Cotonou, Benin; b London School of Hygiene and Tropical Medicine, Keppel St, Bloomsbury, London WC1E 7HT, UK; c MRC Wits Rural Public Health and Health Transitions Research Unit (Agincourt), Faculty of Health Sciences, University of the Witwatersrand, Johannesburg, South Africa; d Department of Intensive Care and Anesthesia, Point G Teaching Hospital, Bamako, Mali; e Infectious disease and tropical medicine Unit, University Teaching Hospital of Point-G, Bamako, Mali; f Service of Pneumology of the University Teaching Hospital of Point-G, Bamako, Mali; g Department of Medicine, Infectious diseases, Lausanne University Hospital and University of Lausanne, Lausanne, Switzerland; h Laboratory for Intelligent Global Health and Humanitarian Response Technologies, EPFL - École Polytechnique Fédérale de Lausanne, Lausanne, Switzerland; i Johns Hopkins University, Bloomberg School of Public Health, 615 N Wolfe St, Baltimore, MD 21205, USA; j University of California, Davis, 4860 Y St., Suite 2300, Sacramento, CA 95817, USA

**Keywords:** Lung ultrasound, point-of-care diagnosis, tuberculosis, interstitial syndrome

## Abstract

Lung ultrasound (LUS) is increasingly used as a diagnostic tool in respiratory medicine, particularly in resource-limited, tuberculosis (TB)-endemic settings. Sonographic interstitial syndrome (IS), characterized by B-line artefacts, has proven valuable in diagnosing cardiogenic pulmonary edema, pneumonia, and chronic interstitial lung disease. However, in TB-endemic areas, its interpretation remains challenging due to overlapping pathologies. This illustrated review explores the diagnostic and prognostic relevance of IS across three clinical contexts: acute respiratory distress, chronic interstitial lung disease, and pulmonary TB. We review current evidence and discuss illustrative cases from TB-endemic settings to highlight sonographic pattern variations and guide clinicians in recognizing key diagnostic features and common pitfalls. IS, while inherently non-specific, gains diagnostic value when contextualized within the patient's background and within additional sonographic findings such as pleural line abnormalities, consolidations, and altered lung sliding. Specific TB manifestations—including miliary TB, cavitary TB, HIV-associated TB, and post-TB sequelae—demonstrate diverse IS presentations. Accurate interpretation requires understanding disease-specific patterns, using appropriate scanning techniques, and adhering to standardized protocols. Greater awareness of IS variations among clinicians can improve diagnostic accuracy and clinical decision-making in TB-endemic regions.

## Highlights

• Lung ultrasound allows rapid bedside detection of interstitial syndrome where radiography is limited in TB-endemic settings.
• In TB-endemic settings, clinical context, pleural irregularities, and small subpleural consolidations improve interpretation.
• This illustrated review compares sonographic patterns across acute respiratory distress, interstitial lung disease, and pulmonary TB.

## 1 Introduction

In the nineties, it was generally thought that lung ultrasound (LUS) was impossible because of air reflecting the ultrasound bundle. This creates a reverberation and shadow artefact and lead clinicians to the common belief that “air is the enemy”. [1] Today, and particularly boosted by the COVID-19 pandemic, LUS has become an attractive bedside skill for many clinicians. Different devices are available ranging from large classic piezo-electric machines to miniaturized point-of-care piezo-electric or ultrasound-on-a-chip devices plugged in on a smartphone. Different scanning protocols exist ranging from 6 to 14 lung-points scanned depending on the clinical context. [2] LUS can evaluate the periphery of the lung and any pathology in contact with the pleural membrane. Central pathology (e.g., a bronchial tumor) is missed because of the surrounding air artefact. The spectrum of LUS signs ranges from typical artefacts for “dry” pathology such as pneumothorax or chronic interstitial lung disease to anatomically representative images for “wet” pathology such as large lung consolidations, peripheral abscesses or pleural effusions. [3] Due to its availability at the bedside and possibility to combine with cardiac ultrasound, LUS was first used and extensively explored in intensive care and emergency settings. The sonographic interstitial syndrome (IS) in these settings, soon became a well-established aid in the diagnosis of cardiogenic pulmonary edema and later pneumonia. [3], [4], [5] The use of LUS gradually broadened to the outpatient setting in internal medicine disciplines such as pneumology and rheumatology. Here, the sonographic IS is of particular interest and under investigation for the follow-up of patients with interstitial lung disease. [6] In the field of pulmonary tuberculosis (TB) diagnostics, LUS is a relatively new player. [7], [8], [9] While its diagnostic performance seems promising for pulmonary TB [10], [11], [12], the significance of the sonographic IS in TB endemic settings remains unclear.

We aim to provide illustrated insights for clinicians, whether novice or experienced lung sonographers, on the sonographic IS in TB endemic regions building on lessons learnt from three diverse clinical settings: 1) Acutely ill patients with cardiogenic pulmonary edema and pneumonia; 2) chronically ill patients with interstitial lung disease and 3) clinical care and recent research on patients with pulmonary TB.

## 2 Definition of the sonographic interstitial syndrome

**Normal air-filled lung is characterized by A-lines** horizontal, repeating ultrasound artefacts rise from ultrasound waves bouncing repeatedly between the probe and pleural line/air interface, best visualized with a linear probe perpendicular to the pleura. The sonographic interstitial syndrome (IS) however is charcaterized by B-lines. It is defined as the presence of at least 3 B-lines between two ribs in a single scan. B-lines patterns may differ according to their distribution (focal, multifocal, diffusely homogenous, or inhomogeneous), intensity and combination with other sonographic signs such as pleural effusions, subpleural consolidations or diminished lung sliding. [13], [14] A B-line is an artefact thought to arise from fluid in the lung interstitial space, creating a hydro-aerial interface. B-lines have seven defining characteristics: a comet-tail shape, arising from the pleural line, hyperechoic, well defined, extending indefinitely, erasing A-lines and moving along with lung sliding (if lung sliding is present). It is important to distinguish the B-line artefacts from *Z*-lines (interference artefacts) which are ill-defined lines arising from the pleural line but that not erase A-lines and do not extend indefinitely. They are estimated to be present in 80% of healthy persons and do not have clinical significance. [5], [14] A dense, confluent pattern of B-lines (so-called “white lung”) reflects more severe interstitial or alveolar involvement, such as in cardiogenic pulmonary edema or ARDS.

## 3 Interstitial syndrome in specific disease states

### 3.1 Acutely ill patients with undifferentiated respiratory syndrome

a) Cardiogenic pulmonary edema

*Sonographic features.*

**Fig. 1:**
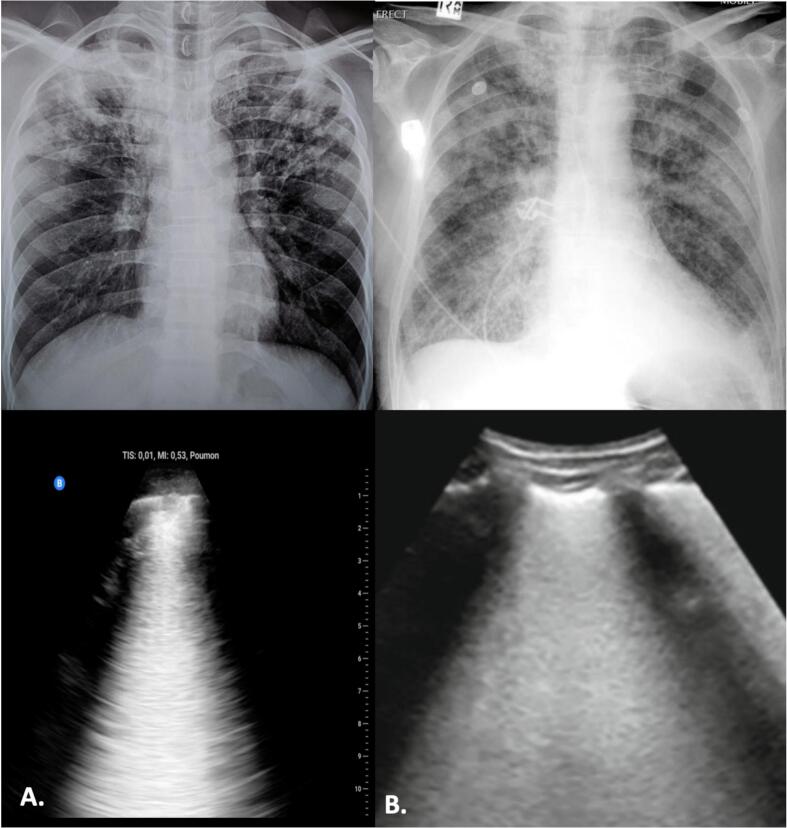
Confluent B-lines or “white lung”. Part A shows a white lung seen in the right apex in a lung mode preset with an ultrasound-on-a-chip device, 10 cm depth. Note the subpleural hypo-echoic consolidation associated. The patient was diagnosed with confirmed pulmonary TB in Benin. Part B. also shows white lung, this time with a normal pleural line and associated to diffuse B-lines in a symmetrical distribution. This patient has pulmonary edema.

In cardiogenic pulmonary edema, high capillary pressures cause protein-poor fluid to transfer out of the capillaries into the lung interstitium and finally into the alveolar spaces. [15] On LUS, this process is reflected by multifocal symmetrically distributed B-lines over both lungs, more marked in the anterior inferior and lateral inferior quadrants and often associated with bilateral (small) pleural effusions. In the absence of other underlying lung pathology, the pleural line appears thin and regular (Fig. 1). [5]

*Diagnostic & prognostic performance.*

LUS has excellent diagnostic performance for detecting cardiogenic pulmonary edema in the critically ill with a pooled sensitivity of 95% (95% confidence interval (CI) 88–98) and a specificity of 91% (95% CI 88–94). [16]

Regarding its prognostic value, the number of B-lines rapidly decreases (within 2 to 3 h) in response to diuretic therapy. In acute heart failure, the presence of more than 15 remaining B-lines on a full anterior lateral lung scan pre-discharge indicates a 5-fold risk (HR 5.5 95%CI 2.24–13.80) for death or readmission. [17]

b) Pneumonia

*Sonographic features.*

In pneumonia, intruding organisms trigger the resident macrophages of the lung. The macrophages engulf these pathogens and trigger both cellular and humoral defense mechanisms and activate complement. This causes inflammation and capillary leaks which leads to protein-rich fluid accumulating in the interstitial space and alveoli. [18] Early on in this process, LUS can show subtle changes including a focal IS, pleural line changes (interruptions, thickening), while later in the process a sonographic alveolar syndrome (e.g., large consolidation in bacterial lobar pneumonia) or a diffuse IS in viral interstitial pneumonia becomes clear. [19] Hence, according to timing of presentation and underlying etiology, pneumonia can take many sonographic forms and the sonographic definition of pneumonia is subject of debate. The BLUE protocol [5] and international expert consensus recommendations [20] propose a broad definition where lung consolidation, a mixed pattern of A-lines and a focal IS or B-lines and absent lung sliding all suggest pneumonia, whereas other authors are more restrictive and use only an alveolar syndrome. This spectrum of definitions implies an inherent trade-off between sensitivity (better for the broader definitions) and specificity (better for the stricter definitions). [21]

*Diagnostic & prognostic performance.*

Overall, when integrated into the routine clinical pathway, lung ultrasound (LUS) demonstrates excellent diagnostic performance for detecting pneumonia of all etiologies. A meta-analysis of 14 studies reported an area under the curve (AUC) of 0.948 for the overall diagnostic accuracy of LUS. The 95% confidence interval (CI) for the pooled estimates indicates a sensitivity of approximately 0.85–0.95 and a specificity of 0.75–0.90. When pneumonia is defined as either consolidation or focal IS, LUS achieves its highest sensitivity at 0.96 (95% CI: 0.90–0.98). In contrast, using a stricter definition of consolidation alone reduces sensitivity to 0.82 (95% CI: 0.74–0.88). Conversely, specificity decreases to 0.74 (95% CI: 0.55–0.87) when the broader definition including interstitial syndrome is applied, compared to a higher specificity of 0.94 (95% CI: 0.85–0.98) when only consolidation is considered. [21] Recent experience with COVID-19 has shown that its sonographic presentation is highly variable, ranging from normal lung findings to diffuse acute respiratory distress syndrome (ARDS). LUS demonstrated a clinically useful pooled sensitivity of 86.9% (95% CI: 80.9%–91.3%) for diagnosing COVID-19 pneumonia in the emergency setting, although the specificity was lower, at 62.4% (95% CI: 49.4%–73.9%). [22]

In selected populations, LUS demonstrated a sensitivity of 94.1% and specificity of 84.8% for H1N1 pneumonia. [23] LUS as a stand-alone test cannot adequately distinguish between viral and bacterial pneumonia. However, in children with community acquired pneumonia, distinct sonographic patterns have been found. Compared to bacterial pneumonia, consolidations seen in viral pneumonia are significantly smaller (15 mm vs. 30 mm *p* < 0.001), multifocal (65.4% vs. 17.3% p < 0.001) and bilateral (51.9% vs. 8.0%, p < 0.001). [24]

For prognosis, using a normalized LUS score taking into account the severity and extent of lung involvement has demonstrated clinical usefulness for the early risk stratification in patients with COVID-19 presenting to the emergency department with an AUROC of 0.80 (95% CI, 0.68–0.92) to discriminate between outpatient management and hospitalization. [25] In pediatrics, LUS scores can inform the clinical course of bronchiolitis by predicting ICU admission, the duration of hospitalization and the need for respiratory support. [26]

### 3.2 Chronically ill patients with interstitial lung disease

#### 3.2.1 Sonographic features

In chronic interstitial lung disease, the lung interstitium (defined as the space between the capillary endothelium and alveolar epithelium) thickens and eventually scars (e.g., pulmonary fibrosis). [27] This process is also reflected by B-line artefacts on LUS. Where it is clear that in itself water, inflammatory or connective tissue, B-lines are indistinguishable, the aspect of the pleural line, distance between the B-lines and diaphragm mobility can give further clues. First, a small single center study suggests a fragmented irregular pleural line is seen more in interstitial lung disease whereas in cardiogenic pulmonary edema, the pleural line mostly appears continuous. [28] Second, it has been suggested that in interstitial lung disease the distance between B-lines correlates with disease severity on chest CT. Dense B-lines (with a distance of <3 mm between each) or confluent B-lines (Fig. 1) correlate with ground glass opacity whereas more spaced B-lines (distance of at least 7 mm) correlate with the further end of the disease spectrum such as fibrosis and honey combing. The distance between B-lines also seems to inversely correlate with pulmonary function values. [29] Third, reduced diaphragm mobility during deep breathing, even though challenging to measure in routine practice, is observed in patients with interstitial lung disease as compared to healthy controls. [30] Importantly, LUS has no clinically useful role in detecting COPD or other rare cystic interstitial lung diseases as the air-filled cysts will cause reverberation artefacts, resulting in A-lines as seen in an otherwise normal dry lung. [31]

#### 3.2.2 Diagnostic & prognostic performance

Regarding the diagnostic value of LUS for interstitial lung disease, The European Respiratory Society (ERS) has recommended further research given the small size, monocentric nature and heterogenous methods of previous studies. [6]

In response to this statement, the AURORA study was launched. This multicenter, cross-sectional study of patients with rheumatoid arthritis and respiratory symptoms seeks to determine the diagnostic performance of LUS as compared to high-resolution computed tomography as a reference standard. [32]

Regarding prognosis, adding a cardiopulmonary ultrasound score based on number of B-lines and right ventricular end-diastolic dimension to a standard clinical score (Gender-Age-Physiology score) significantly improves its predictive value for mortality risk (c statistics 0.90 vs 0.76, *p* = 0.018). [33]

### 3.3 Insights for the sonographic IS in pulmonary tuberculosis

#### 3.3.1 Sonographic features

##### 3.3.1.1 Miliary TB

**Fig. 2:**
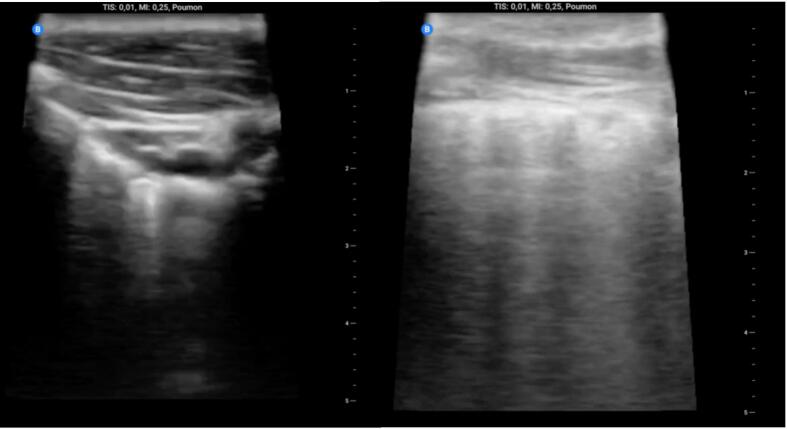
**(INSERT VIDEOLINK):** Left; sonographic image in lung mode at 5 cm (as a linear probe) depth of TB sequelae in the right apex. Note the broken irregular pleural line with loss of lung sliding and the lung pulse visible on B-mode due to massive destruction of the lung parenchyma. Right: sonographic image in lung mode (as a linear probe) at 5 cm depth of a miliary infiltrate extending to the lung periphery. Note almost regularly spaced B-lines with preserved lung sliding and the presence of granular subpleural hypoechoic consolidations.

In miliary TB, there is a massive lymphohaematogenous spread of *Mycobacterium tuberculosis* from a pulmonary or extrapulmonary focus and embolisation to the vascular beds of various organs. Histopathological images typically demonstrate granulomatous inflammation. In the lungs, chest X-ray correspondingly shows a miliary infiltrate, which may not be seen until late in the course of the disease. [34] On LUS, the miliary pattern is seen as multiple rather regularly spaced B-lines and subpleural granularity, sometimes with small subpleural consolidations, seen in almost every quadrant. It is best captured on video or clip (Fig. 2). [2], [35]

#### 3.3.2 TB cavities

A TB cavity is a walled gas-filled space in the lung parenchyma resulting from infection with *Mycobacterium tuberculosis,* subsequent granulomatous abscess formation and central necrosis. Depending on various immune-related host factors, most TB cavities are found in the apical or posterior segments of the superior lobes and less frequently the upper segments of the inferior lobes. [36] As gas-filled structures cause reverberation artefacts, an isolated central cavity in itself cannot be directly visualized on LUS. [8], [37] However, its inflammatory border can result in a surrounding interstitial or alveolar syndrome,including large consolidations,small subpleural consolidations and/or pleural line irregularities). The small proportion of cavities that have fluid in them might be easier to see on LUS.

**Fig. 3:**
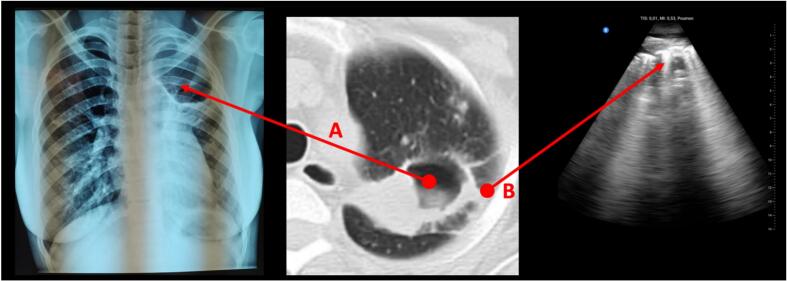
Arrow A; cavities are easily detected on CT-scan and chest X-ray. Arrow B; Gas-filled cavities remain invisible on lung ultrasound because of the reverberation artefact. However, their inflammatory boarders can be detected and can cause an interstitial or alveolar syndrome. Here we note multiple B-lines, a broken and irregular pleural line with subpleural consolidations.

Recent data suggest that interstitial or alveolar syndromes found in the apical region are associated with bacteriologically confirmed TB. This study in symptomatic adults found a significant association between bacteriologically confirmed pulmonary TB and apical consolidations >1 cm (OR 19.17; 95% CI 11.2–32.8; *p* < 0.001), apical subpleural consolidations <1 cm or irregular/broken pleural lines (OR 3.66; 95% CI 2.5–5.3; p < 0.001) and apical B-lines (OR 1.52; 95%CI 1–2.30; *p* = 0.043). [38] Clinicians should be aware of this caveat (Fig. 3).

#### 3.3.3 HIV related TB

TB can develop at any stage of HIV disease. The level of immunosuppression greatly impacts its clinical presentation. In early stages of HIV disease, TB manifests similarly compared to HIV-uninfected persons: a mainly pulmonary disease often with cavitation on chest X-ray and smear positive on sputum. In immunosuppressed patients, more extra-pulmonary disease is seen and pulmonary disease becomes less evident on chest X-ray (lymph node enlargement, minimal parenchymal lesions, miliary pattern) and microbiology (paucibacillary disease resulting in a negative smear). [39] While there is reasonably strong evidence to support the use of pericardial and abdominal ultrasound to detect foci of extra-pulmonary TB, LUS findings for HIV associated pulmonary TB are less well described. [8] A sonographic IS sometimes associated with focal or diffuse small subpleural consolidations, pleural or pericardial effusion might be expressions of TB in the immunosuppressed. [40], [41] *Pneumocystis jirovecii* pneumonia has overlapping sonographic characteristics with diffuse symmetrical B-lines and subpleural consolidations whereas major lung consolidations and pleural effusion should prompt suspicion of pulmonary TB or bacterial pneumonia. [42], [43]

#### 3.3.4 TB sequelae

**Fig. 4:**
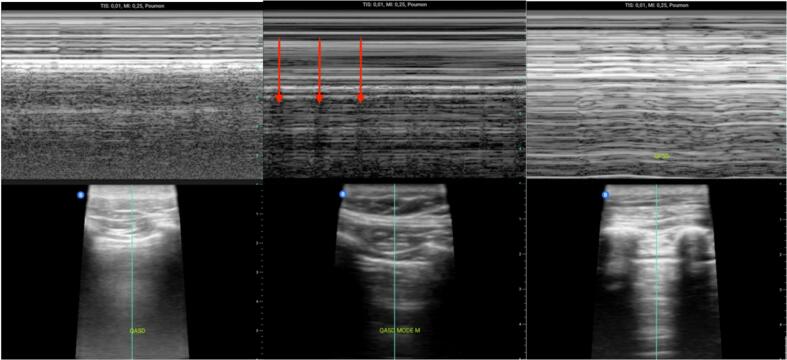
Lung sliding can be evaluated in M-mode asking the patient to take deep breaths. Left; Normal lung sliding results in a sandy beach image on M-mode. Middle; sometimes the lung pulse can be observed as regular (on the cardiac frequency) vertical linear artefacts stopping at the pleural line (red arrows). Vertical artefacts in M-mode passing the pleural line are typically movement artefacts (e.g., respiratory distress and tachypnea). Right; Diminished lung sliding shows a barcode sign on M-mode. The barcode sign in itself is nonspecific and can be observed in various conditions where there is loss of air movement such as emphysema, COPD, pneumothorax or TB sequelae with residual cavities and parenchymal destruction. Note that the presence of lung pulse excludes pneumothorax. It is important to note that the lung apexes physiologically present less lung sliding than the other quadrants. (For interpretation of the references to colour in this figure legend, the reader is referred to the web version of this article.)

**Fig. 5:**
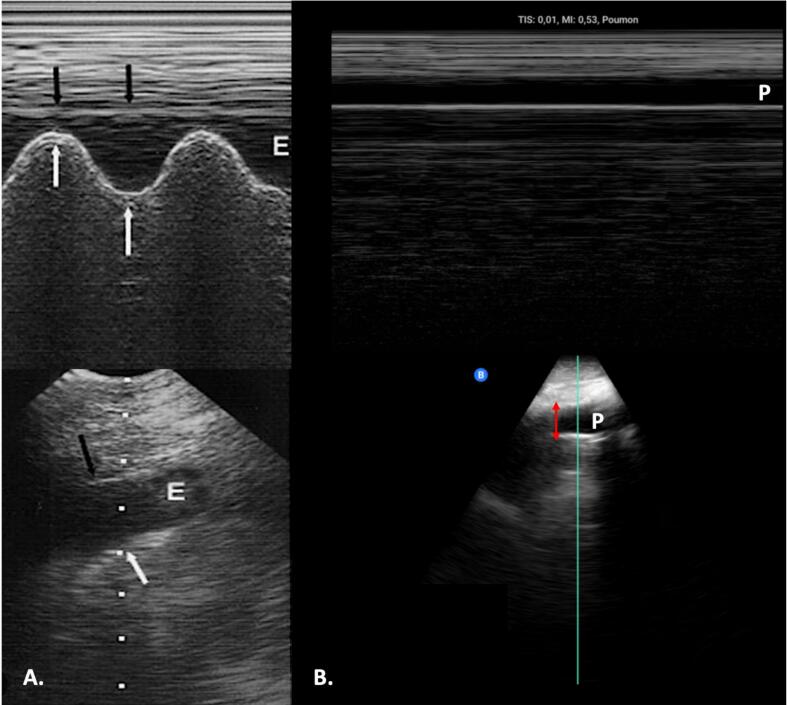
part A. represents a pleural effusion, below in B-mode, above showing the sinusoid sign in M-mode demonstrating the presence of fluid undulating with the beating of the heart. Part B shows a case of post TB thickening of the pleural membrane. Pachypleuritis appears as a well-demarcated hypo-echoic area between the rib cortex and the pleural line (red arrow). On M-mode above, the sinusoid sign is notably absent as there is no effusion involved. *E* *=* *effusion, P* *=* *pachypleura*. Fig. 5 A is adapted from [50].

Even with effective treatment, the majority of patients with pulmonary TB will develop long-term pulmonary sequelae ranging from asymptomatic radiologic lesions to debilitating residual cavities and bronchiectasis in up to half of patients. On spirometry, at least 40% of patients have results out of the normal range, of which over half demonstrate an obstructive syndrome. [44] As compared to chest X-ray, important TB sequelae with residual cavities, calcification, fibrosis, atelectasis and bronchiectasis are more difficult to interpret on LUS and result in a range of signs seen in both IS and alveolar syndromes. However, being a dynamic examination, additional signs can be used. For instance, the lung pulse can provide interesting clues. Lung pulse is a LUS sign where rhythmic movement of the pleural line corresponds to the patient's cardiac activity and is typically looked for in M-mode (movement mode). It indicates that the visceral and parietal pleura are in contact but there is no lung sliding, commonly seen in complete atelectasis or apnea. Its presence rules out pneumothorax in the scanned area. In healthy parenchyma, it is not or only very subtle seen on M-mode and invisible on B-mode. Where there is important parenchymal destruction, the lung pulse can be observed in B-mode (Fig. 2). Lung sliding can also be diminished or even absent, seen on B-mode as a still pleural line, and on M-mode as a barcode sign (Fig. 4). Another hint is pachypleuritis. Thickening of the pleura can arise from various inflammatory, infectious or neoplastic processes. After TB pleurisy, an estimated 10 to 72% develop pleural thickening. [45] On LUS, pachypleuritis appears as a well-demarcated hypo-echoic area between the rib cortex and the pleural line. The operator might first think of a pleural effusion, but on M-mode, the sinusoid sign (typically present when liquid content undulates with the beating of the heart) will be notably absent (fig. 5). Calcifications might be present, appearing as discontinuous hyperechogenic lines casting shadow cones. [46] The secondary loss of diaphragm mobility as described in chronic interstitial lung disease remains unstudied.

#### 3.3.5 Diagnostic and prognostic performance of LUS for pulmonary TB

The diagnostic performance of LUS for pulmonary TB seems promising with reported sensitivities ranging from 73 to 100%. [41], [47] A recent large prospective cohort study conducted at a tertiary center in Benin evaluated 504 symptomatic adult patients and demonstrated excellent diagnostic performance of lung ultrasound (LUS) for tuberculosis. Using human expert image interpretation, LUS achieved a sensitivity of 0.90 (95% CI: 0.89–0.92) and a specificity of 0.61 (95% CI: 0.54–0.67), with an area under the receiver operating characteristic curve (AUROC) of 0.84 (95% CI: 0.82–0.85). When interpretation was performed using artificial intelligence, the sensitivity increased slightly to 0.91 (95% CI: 0.89–0.92), and specificity improved substantially to 0.85 (95% CI: 0.81–0.90), with a higher AUROC of 0.93 (95% CI: 0.92–0.95) (*p* < 0.001). The study found a significant association between bacteriologically confirmed TB and apical consolidations >1 cm (OR 19.17; 95% CI 11.2–32.8; *p* < 0.001) apical subpleural consolidations less than 1 cm or irregular/broken pleural lines (OR 3.66; 95% CI 2.5–5.3; p < 0.001) and apical B-lines (OR 1.52; 95%CI 1–2.30; *p* = 0.043). Across all quadrants, TB was also associated with consolidations >1 cm (OR 15.20; 95% CI 9.7–23.8; p < 0.001), small subpleural consolidations or irregular/broken pleural lines (OR 4.36; 95% CI 2.3–8.1; p < 0.001). [38]

## 4 Lessons learnt and illustrated insights

As different pathophysiological processes can generate B-lines (fluid accumulation below the visceral pleura and in the interstitium, thickened interlobular septa or lung deflation), it is impossible to distinguish them as B-lines related to either water or inflammatory fluid. [48] The diagnostic performance of the sonographic IS for cardiogenic pulmonary edema and pneumonia has been derived from selected populations with a high pre-test probability for each relative condition and most often in regions with low TB incidence. In reality, the IS on itself is rather nonspecific and has to be placed in each patient's clinical context.

Fig. 1A and B both display a characteristic sonographic “white lung” pattern. Fig. 1A is from a patient presenting with chronic-onset dyspnea in a high tuberculosis (TB) endemic setting. The ultrasound reveals an irregular pleural line and a small hypoechoic small subpleural consolidation. This patient was subsequently confirmed to have pulmonary TB. In contrast, Fig. 1B shows a patient with acute-onset dyspnea, lower limb edema, and multiple cardiovascular risk factors, including arterial hypertension. This patient was from a low TB prevalence setting and was diagnosed with pulmonary edema.

Fig. 2 illustrates two patients, both presenting with chronic-onset dyspnea in a TB endemic region and both demonstrating a B-line pattern on lung ultrasound. Patient A exhibits a focal interstitial syndrome with a broken, irregular pleural line, absence of lung sliding, and the presence of a lung pulse on B-mode—features indicative of TB sequelae. Patient B, on the other hand, shows a diffuse interstitial syndrome with regularly spaced B-lines, preserved lung sliding, and small granular subpleural hypoechoic consolidations, consistent with miliary TB.

**Fig. 6:**
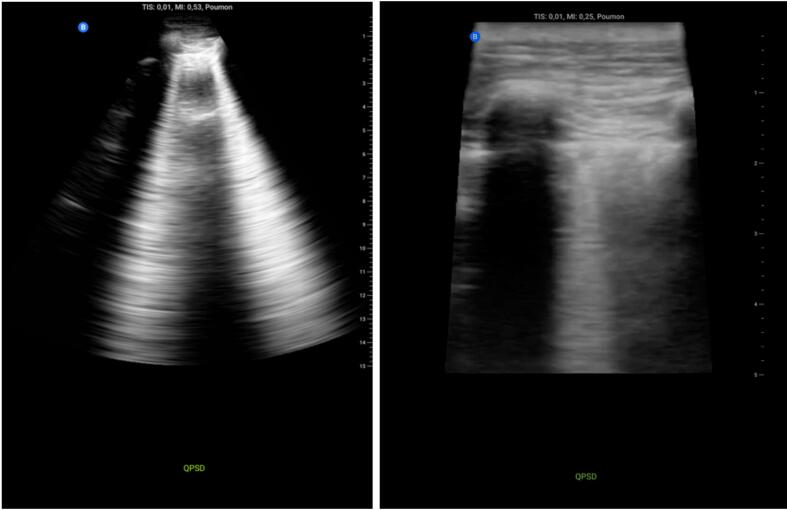
Left; sonographic image in lung mode at 15 cm depth (as a convex or phase-array probe) of two B-lines, note that the detailed aspect of the pleural line is invisible in this view Right; same quadrant viewed in lung mode at 5 cm depth (as a linear probe). Details of the aspect of the pleural line become apparent and we note an irregular pleural line with a hypo-echoic subpleural nodule.

**Fig. 7:**
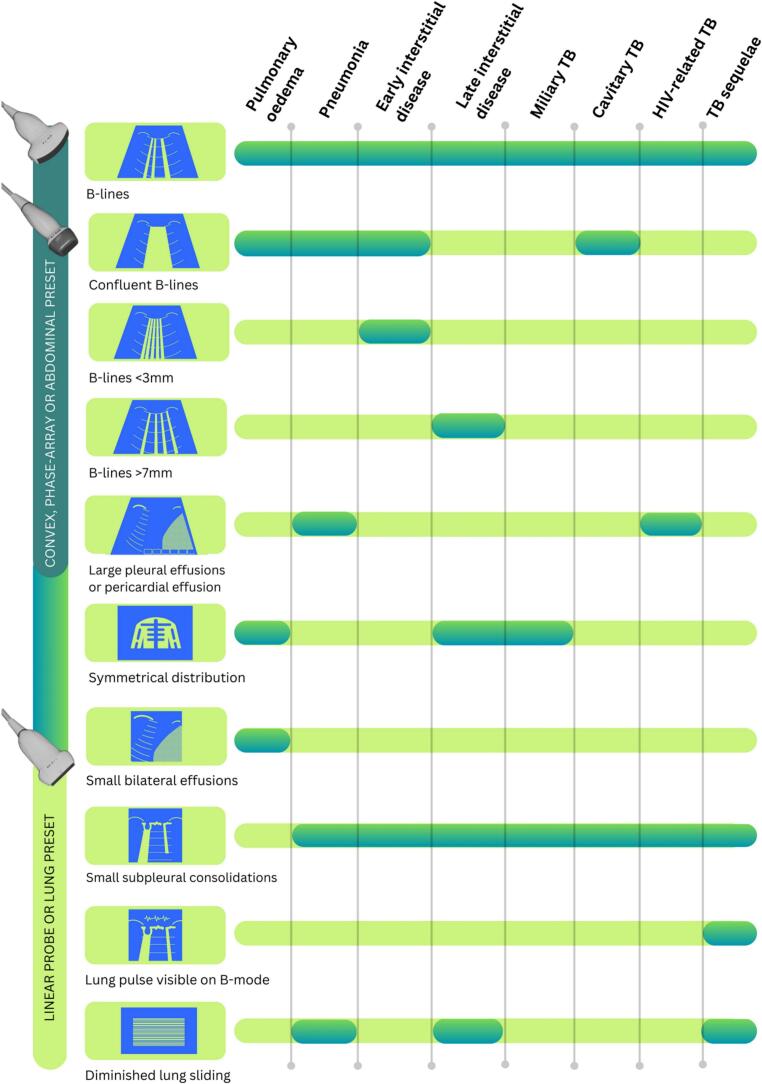
Summary of different sonographic signs that can be associated with the B-line pattern for different clinical syndromes and diseases. Significant overlap exists between signs and sonographic findings must be strictly interpreted within the patient's clinical context and local epidemiologic setting. On the left, different probes are shown (or presets for ultrasound on a chip devices). Findings can be visible with either type of probe/preset but the convex, phase-array or abdominal preset tends to facilitate the view of deeper structures or a quick overview of the type of B-line pattern associated pleural or pericardial effusions, whereas the linear probes or lung preset can help provide a detailed image on the pleural line and might facilitate the detection of small subpleural consolidations or pleural irregularities, lung pulse and lung sliding.

These cases illustrate that subtle differences in the sonographic IS and the presence or absence of other sonographic signs such as the aspect of the pleural line and lung sliding or presence of effusion may give useful clues, when interpretated within their epidemiological and clinical context. However, finding these clues is highly technique and operator dependent. [49] For instance, signs of an interrupted or irregular pleural line with or without small subpleural consolidations are most easily detected with high frequency probes (linear probe; lung preset at 5 cm depth on ultrasound-on-a-chip devices) focusing on the pleural line with a depth of max 5 cm in non-obese patients. On the other hand, a good view on the number and distribution of B-lines is easier at 12–15 cm depth and associated larger pathology (pleural effusions, consolidations) can be missed or is more difficult to interpret on the high frequency images (Fig. 6). While it is still unclear which lung scanning protocols are best adapted for TB endemic regions [2], LUS operators must be aware of the influence of different probes and settings on the appearance of the IS and its associated spectrum of overlapping signs (summary Fig. 7). Finally, important clinical questions remain open for further research notably the role of LUS in HIV-associated pulmonary TB and in the follow-up of patients with TB sequelae. Lastly, while some of the subtleties that may help distinguish between these different pathologies is certainly technique and operator dependent, AI might have a role to play here in assisted image interpretation.

## 5 Conclusion

In TB-endemic settings, the sonographic IS presents both a diagnostic opportunity and a challenge. While IS is inherently non-specific, its diagnostic value increases significantly when interpreted within the patient's clinical context and complementary ultrasound signs. By recognizing disease-specific patterns and chosing appropriate scanning techniques, clinicians can better differentiate between overlapping respiratory pathologies. A nuanced understanding of the IS is valuable to improve diagnostic accuracy and patient clinical decision-making in the complex landscape of respiratory syndromes in TB endemic areas.

## Ethics/consent statement

Images were obtained under the TrUST study protocol. This protocol was approved by the local ethics committee for biomedical research of the university of Parakou, Benin on May 18, 2021; The study followed the Declaration of Helsinki. All participants provided written informed consent. For participants who were unable to read or write, consent was obtained with the assistance of a trusted representative.

## Author Contribution

AW, SM and VS conceptualized this narrative. VS wrote the first draft and supervised the final manuscript. VS developed the summary figure. AW, NBB, MF, TB edited and reviewed the manuscript. GA and WA validated the image reviews. Image analysis was performed by AH, JDD, SAB, YC, KO, SM.

## CRediT authorship contribution statement

**Ablo Prudence Wachinou:** Writing – review & editing, Writing – original draft, Conceptualization. **Matthew Fentress:** Writing – review & editing. **Jacques Du Toit:** Investigation, Formal analysis. **Aboudou Hada:** Investigation, Formal analysis. **Gildas Agodokpessi:** Validation, Project administration. **Seydina Alioune Beye:** Investigation, Formal analysis. **Yacouba Cissoko:** Investigation, Formal analysis. **Khadidia Ouattara:** Investigation, Formal analysis. **Shabani Majaliwa:** Validation, Conceptualization. **Thomas Brahier:** Writing – review & editing. **Noémie Boillat-Blanco:** Writing – review & editing. **Véronique Suttels:** Writing – review & editing, Writing – original draft, Visualization, Conceptualization.

## Declaration of competing interest

The authors declare that they have no known competing financial interests or personal relationships that could have appeared to influence the work reported in this paper.

## Glossary

**Lung Ultrasound (LUS)**: A bedside imaging technique that uses high-frequency sound waves to evaluate lung pathology, particularly in the pleural and subpleural regions.
**Sonographic Interstitial Syndrome (IS)**: A lung ultrasound pattern defined by the presence of at least three B-lines between two ribs in a single scan area, generally thought to reflect interstitial involvement.
**B-lines**: Vertical, hyperechoic artefacts that originate from the pleural line and extend indefinitely to the bottom of the ultrasound screen without fading. They move with lung sliding and are associated with interstitial fluid or thickening.
**A-lines**: Horizontal reverberation artefacts that represent normal aerated lung. They indicate the presence of air between the pleura and the ultrasound probe.
**Pleural Line**: The sonographic representation of the interface between the visceral and parietal pleura. Its appearance can vary (smooth, irregular, thickened) depending on the underlying pathology.
**Lung Sliding**: The horizontal shimmering movement of the pleural line seen in real-time B-mode, indicating that the visceral and parietal pleura are in contact and moving with respiration.
**Lung Pulse**: A rhythmic movement of the pleural line in synchrony with cardiac activity, observed when lung sliding is absent but the pleural layers are still in contact. Best looked for in M-mode It helps rule out pneumothorax. Can be flagrant and visible even in B-mode in the case of massive parenchyme destruction such as TB sequelae.
**Subpleural Consolidation**: A hypoechoic or tissue-like area beneath the pleura indicating localized lung pathology, such as infection, inflammation, or infarction.
**Pachypleuritis**: Thickening of the pleural membranes, often due to chronic inflammation or infection, such as tuberculosis. On ultrasound, it appears as a hypoechoic band between the rib cortex and pleural line. On M-mode the sinusoid sign is notably absent.
**Barcode Sign**: An M-mode ultrasound pattern with horizontal lines throughout, indicating absent lung sliding, seen in conditions such as pneumothorax, COPD or pleural adhesion.
**Sinusoid Sign**: An M-mode finding within pleural effusions, showing fluid movement in synchrony with cardiac or respiratory motion, helping differentiate free fluid from pleural thickening.
**Point-of-Care Ultrasound (POCUS)**: The use of portable ultrasound at the patient's bedside for real-time diagnosis and monitoring in clinical practice.
**High-frequency Linear Probe**: An ultrasound transducer with high resolution used for superficial structures, ideal for evaluating the pleural line and subpleural changes.
**Convex or Curvilinear Probe**: A lower-frequency ultrasound transducer that provides a wider and deeper field of view, useful for assessing lung fields and deeper pathology.

## Appendix A Supplementary data

Supplementary Video 1 Lung ultrasound showing a discrete B-line arising from the pleural line, extending to the far field without fading, and moving synchronously with lung sliding, consistent with interstitial syndrome.

Supplementary Video 2 Lung ultrasound showing multiple coalescent B-lines arising from the pleural line and extending to the far field without fading, producing a white-lung pattern consistent with interstitial syndrome.
